# Study on Topology Optimization Design, Manufacturability, and Performance Evaluation of Ti-6Al-4V Porous Structures Fabricated by Selective Laser Melting (SLM)

**DOI:** 10.3390/ma10091048

**Published:** 2017-09-07

**Authors:** Yangli Xu, Dongyun Zhang, Yan Zhou, Weidong Wang, Xuanyang Cao

**Affiliations:** Institute for Laser Engineering, Beijing University of Technology, Pingleyuan No. 100, Chaoyang District, Beijing 100124, China; ylxubjut@outlook.com (Y.X.); zy123zhou@163.com (Y.Z.); 18739823256@163.com (W.W.); caoxuanyang@126.com (X.C.)

**Keywords:** selective laser melting, topology optimization, porous structure, elastic modulus, compression behavior, stability

## Abstract

The combination of topology optimization (TOP) and selective laser melting (SLM) provides the possibility of fabricating the complex, lightweight and high performance geometries overcoming the traditional manufacturing “bottleneck”. This paper evaluates the biomechanical properties of porous structures with porosity from 40% to 80% and unit cell size from 2 to 8 mm, which are designed by TOP and manufactured by SLM. During manufacturability exploration, three typical structures including spiral structure, arched bridge structure and structures with thin walls and small holes are abstracted and investigated, analyzing their manufacturing limits and forming reason. The property tests show that dynamic elastic modulus and compressive strength of porous structures decreases with increases of porosity (constant unit cell size) or unit cell size (constant porosity). Based on the Gibson-Ashby model, three failure models are proposed to describe their compressive behavior, and the structural parameter *λ* is used to evaluate the stability of the porous structure. Finally, a numerical model for the correlation between porous structural parameters (unit cell size and porosity) and elastic modulus is established, which provides a theoretical reference for matching the elastic modulus of human bones from different age, gender and skeletal sites during innovative medical implant design and manufacturing.

## 1. Introduction

Metallic implants are commonly used to replace human bones that are defective or lost due to oncotherapy, traffic accidents and so on. However, these metallic implants manufactured by traditional methods should be given more attention, because they are made out of dense materials and cannot achieve the transport of water and nutrients [1]. Also, it possibly causes a stress shielding phenomenon [2] due to their higher elastic modulus than natural human bone, leading to implant fracture [3], bone necrosis and other problems. Porous structures are innovative materials, in which they have higher porosity and correspondingly reduce their elastic modulus. With the development of technology, more and more people hope that the biomechanical properties of porous structure can be customized by their designed unit cell using advanced design methods and then manufactured by advanced technology. However, how to design these complex porous structures and then precisely and successfully fabricate them is still a problem we have to confront. Exploring how to design and manufacture the innovative porous structures for medical application and to evaluate their biomechanical properties is necessary. 

Titanium implants with dedicated porous structures have great advantages because of the improvement of permeability and increase of ingrowth to bone cells [4]. But for surgical application, the effect of some parameters such as porosity, pore size, and pore connectivity of porous structures on their biomechanical properties is not clear, and should be intensively investigated and evaluated. Although the main design methods of porous structures, including CAD(Computer Aided Design)-based design [5], image-based design [6] and implicit surface modeling [7] have been developed in recent years, either their compressive strength cannot meet the medical application standard or their elastic modulus distribution shadow is relatively narrow, which makes implants not easily match of human bone. Here, topology optimization (TOP) is used to construct geometries and mechanical parts for achieving lightweight and at the same time the maximum global stiffness. Some scholars converted topology optimized unit sell structure as microstructure of femoral implant, and the numerical simulation with different porosity and unit cell size showed the implants with gradient porous structure had better bone resorption, but no related performances were reported [8]. Xiao [9] optimized the porous structure with topology to achieve the biomaterial and manufactured it using SLM, but didn’t research its mechanical properties. Because material distribution in geometries designed by TOP depends on stress clouds, which causes the optimized geometries to become irregular and complicated, traditional manufacturing methods such as casting and forging encounter severe challenges. Selective laser melting (SLM) technology, as one of the layer-wise manufacturing methods, extends its manufacturing limit to the geometry with tiny intern shapes. However, the manufacturability of geometries optimized by TOP also needs to be investigated, and the evaluation for their biomechanical properties needs to be discussed. 

Although SLM technology extends its manufacturing limit to the geometry with tiny intern cavities and holes, the geometry with overhangs at the angle of 30° to the horizontal cannot be manufactured because of not enough support from underlayer powder during the SLM process, which should be investigated firstly because of its difference in materials, geometries and type of SLM machine. Many researchers [10,11,12,13] have discussed the manufacturability of metallic parts using SLM under their special investigation condition. Di Wang exhibited the results for manufacturing limits of parts from 316L stainless steel with different geometrical features including sharp corners, inclined plane, holes, cylinders, thin walls and so on, indicating the design constraints of SLM, such as the minimum manufacturing resolution, the reliable building angles and the optimization of the surface quality [10]. Maciej Mazur [11] has given out the manufacturing limits Ti-6Al-4V cantilever strut elements through the manufacturing of porous lattice structures, strut diameter of 0.3 mm and inclination angle of 30°. Ihar Yadroitsau reported a series of works about the manufacturing analysis of the SLM metallic parts, in which 140 μm-thick walls is a manufacturing limitation [12] and more complex inner structures manufactured by SLM is investigated for functional components [13]. The above investigation helps to make up for the shortcomings of designs based on their research, which provides the possibility to precisely and successfully manufacture the designed geometries and further evaluate their biomechanical properties. 

Ti-6Al-4V possesses excellent biomechanical properties, biocompatibility and corrosion resistance, is considered an important innovative material of porous structure for medical implants, and its performance has correspondingly been paid extensive attention recently. The biomechanical properties of porous structures are determined significantly by unit cell size, porosity, material and manufacturing characteristics so well. As medical implants, porous structures fabricated from Ti-6Al-4V could reduce elastic modulus to make it match that of human natural bone, and at the same time they should be able to bear the load of the corresponding parts from the body. However, the decrease of elastic modulus simultaneously causes the decrease compressive resistance. An investigation to evaluate the mechanical performance of porous structures such as compressive behavior or stiffness and elastic modulus should be carried out. Cheng [14] fabricated two kinds of Ti-6Al-4V porous structures based on stochastic foams and reticulated mesh with different densities by Electron Beam Melting (EBM); a fluctuating yield platform in the stress-strain test appeared and indicated that the failure model of Ti-6Al-4V porous structures follows the elastic-brittle foam model reported by Gibson and Ashby [15]. Maciej Mazur [11] fabricated six kinds of porous structures with different unit cells designed by topology, and their stress-strain curves showed that their compressive behavior has a similar tendency with only a slight difference. Similar results are also reported by Sing et al., whose unit cell is a square pyramid and truncated cube and octahedron designed by CAD software [16]. These researches have achieved great progress, but these results are scattered, not systematic enough to evaluate the biomechanical properties of porous structures, and the majority of porous structures were not designed by TOP.

This paper designs porous structures using TOP under the condition of human skeletal stress, obtains a unit cell and constructs a series of porous structures with porosity from 40% to 80% and unit cell size from 2 to 8 mm, and then their manufacturability for SLM is investigated. The properties such as compression strength and dynamic elastic modulus are measured. For the manufacturing limit investigation, three typical internal microstructures are abstracted out of porous structures and their manufacturing limits are investigated. The compressive behavior is explained and three failure models of porous structures are proposed; at the same time, the stability of porous structures is evaluated, in which the Gibson-Ashby model plays an important role. Finally, a numerical model for the correlation among elastic modulus, porosity and unit cell size is established, which indicates the methods for parameter choice during the design and manufacturing of porous structures for medical applications. 

## 2. Materials and Methods 

### 2.1. Equipment and Materials

The SLM specimens are made on EOS M280 equipment (manufactured by the German Company EOS, Freiberg, Germany), which is equipped with a Yb-fibre 400 W laser with a focal spot diameter of 100 µm. The maximum scanning speed and layer thickness distribution are 7000 mm/s and 20–50 µm, respectively. The maximum building volume (W × D × H) is 250 mm × 250 mm × 325 mm. The build chamber was flown with argon to avoid oxidation and nitriding of liquid metal in molten pool and the density of specimens can be achieved above 98%. EOS Titanium Ti-6Al-4V (a titanium alloy powder) was used and average size of the powder particles was 45 µm with an apparent density of 4.41 g/cm^3^; the nominal composition of the gas atomized powder was Ti (85.7 wt %), Al (6.75 wt %), V (4.5 wt %), the ingredients (balance).

### 2.2. Design of Unit Cell and Porous Structures Using Topology Optimization

**Figure 1:**
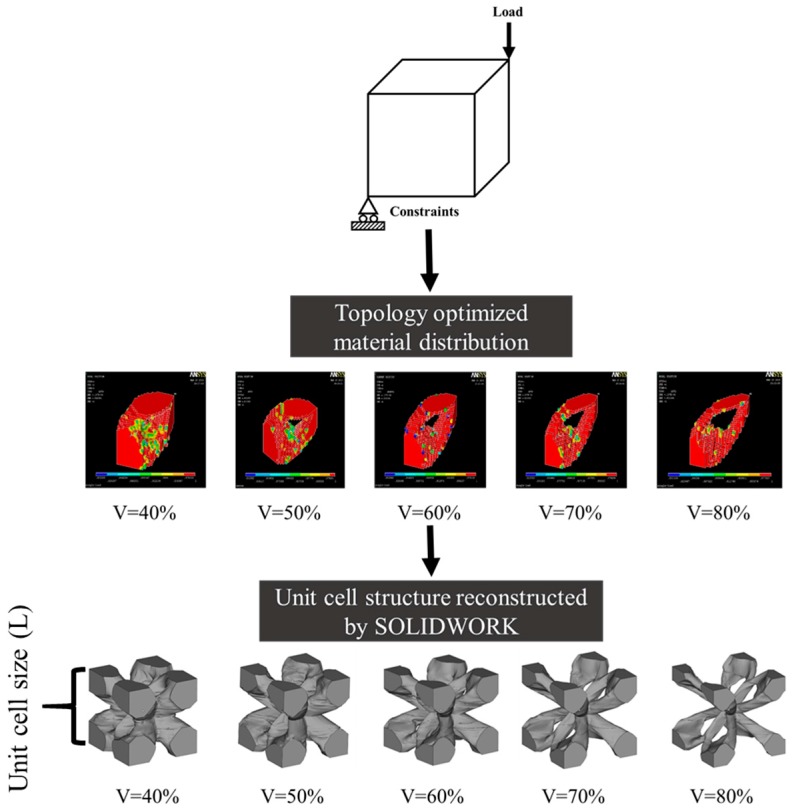
Process of topology optimization (TOP), obtaining the 1/8th unit cell and unit cell after Boolean operation with different porosity.

TOP is used to construct a structure for achieving lightweight and, at the same time, maximum global stiffness. Construction method of TOP porous structure stems reported by Gilbert Chahine [17]. In the process of structure optimization as shown in Figure 1, the structure model can be simplified as a cuboid, of which the vertex at the lower left corner is fixed as the fulcrum of force and its diagonal opposite vertex is subjected to a force. Material model was established with an elastic modulus of 110 GPa and a Poisson’s ratio of 0.33, respectively, which corresponds to the mechanical properties of Ti-6Al-4V. The objective function is the maximum structure stiffness and the constraint function is porosity. The constraint function is changed by porosity from 40%, 50%, 60%, 70% and 80%, and the stress clouds describing material distribution corresponding to different porosity are obtained. SOLIDWORK is used to transform the profile of stress clouds in Z-axis into the geometry model, and a 1/8th unit cell in open cell configuration with different porosity is obtained. Magics 21.0 software is used to repair and smooth the surface of the 1/8th unit cell. A complete unit cell is obtained by Boolean operation of the 1/8th unit cell. For the complete unit cell whose silhouette is like a cube, and the length of a side of complete unit cell is defined as “unit cell size” labeled in the Figure 1. According to the primary theory of topology optimization, the array material obtained by stacking unit cells has almost the same mechanical properties as the designed cells.

### 2.3. Compression Test 

**Figure 2:**
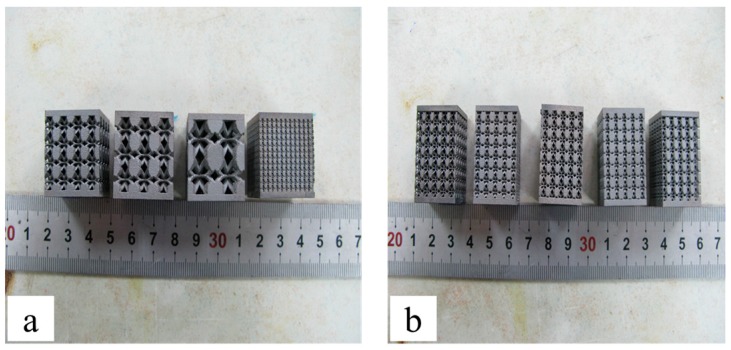
Two group specimens of porous structures manufactured by selective laser melting (SLM) for compression tests: (**a**) specimens with a unit cell size of 2 mm and different porosity of 40%, 50%, 60%, 70% and 80%; (**b**) specimens with a porosity of 60% and different cell sizes of 1, 2, 3, 4 and 6 mm.

Two group specimens of porous structures for compression tests are manufactured by SLM. Each specimen is filled with unit cell with different size and porosities in a certain space. The first group has a porosity of 60% and different unit cell sizes of 1, 2, 3, 4 and 6 mm, respectively. Another group has a unit cell size of 2 mm and different porosity of 40%, 50%, 60%, 70% and 80% (Figure 2a,b), respectively. In order to eliminate the residual stress existing inside the specimens, the heat treatment for stress relief with the temperature (800 °C + 4 h) and furnace cooling is carried out. All the specimens are cut down by wire cutting machine and then processed by sand blasting machine. The compression tests are carried out using the universal material specimen machine (ZWICK/ROELL Z050, Kennesaw, KY, USA) at room temperature. To get detailed information about the process of structures deforming and crushing, the compression tests are monitored by a digital camera.

### 2.4. Calculation of Porosity and Measurement of Dynamic Elastic Modulus

**Figure 3:**
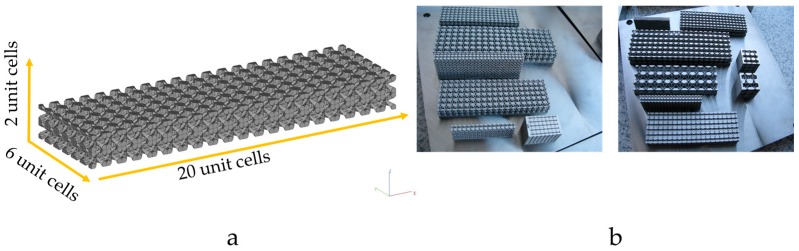
(**a**) Schematic diagram of porous lattice structure; (**b**) porous structures with different unit cell size and porosity manufactured using SLM.

To measure the dynamic elastic modulus of porous structure, each specimen has the same numbers of unit cells and their distribution in space is 6 × 20 × 2 unit cells, which partially shown in Figure 3. Twenty-five porous lattice structures with different porosity of 40%, 50%, 60%, 70% and 80% and unit cell sizes of 1, 2, 3, 4 and 6 mm are constructed based on the array of unit cells by the Boolean operation and manufactured by SLM, and the experimental porosity of the porous structure is obtained by the mass method. The equation is given in the following:

(1) $$P=\left(1-\frac{M}{V\rho_{S}}\right)\times 100\%=\left(1-\frac{\rho }{\rho_{S}}\right)\times 100\%$$

where *P* is porosity of porous structure, *M* is weight of specimen (g) and *V* is spatial volume (including pore) of specimen (cm^3^). The weight is measured by electronic scales and the volume is calculated by measurement values using Vernier calliper (*V* = length × width × height of porous structure). $\rho_{S}$ is the material density of Ti-6Al-4V (i.e., 4.43 g/cm^3^). 

The dynamic elastic modulus of porous structures is determined using resonant frequency and damping analyzer (RFDA) from the IMCE Company (Genk, Belgium). During testing, the measured specimen dissipates its energy into the vibration expressing as a damping sine wave [18]. The vibration induced from a small mechanical impulse of measured specimens is in fact the sum of several resonant frequencies *f_r_*, each of which will dampen according to the energy absorption in the material. For the porous structures in the paper, the vibration modes are well-defined and the dynamic elastic modulus is theoretically given by the equation in the following:

(2) $$E=\;\zeta mf_{r}{}^{2}$$

where *ζ* is a function related to geometrical shape and *m* is the mass of specimen. RFDA measures the dynamic elastic modulus, which may reflect more precisely its inner structural features and stiffness compared with the static elastic modulus from the compressive strength test. 

## 3. Results and Discussion

Topology optimized geometry possesses lighter weight and at the same time better working performances compared with the traditionally designed one. Traditional manufacturing methods, such as casting and forging, encounter severe challenges due to the complexity of topology optimized geometry. Although SLM technology extends its manufacturing limit because of support from the underlayer powder, it still has some manufacturing limits such as the minimum manufacturing resolution, the reliable building angles and the optimization of surface quality, what should be investigated firstly because of its difference in materials, geometries and type of SLM machine. Furthermore, biomechanical properties of Ti-6Al-4V porous structures should be evaluated to see whether they meet that of human bone, and their stability and the relationship between porous structural parameters and elastic modulus will also be discussed.

### 3.1. Manufacturability of Topology Optimization Structure

**Figure 4:**
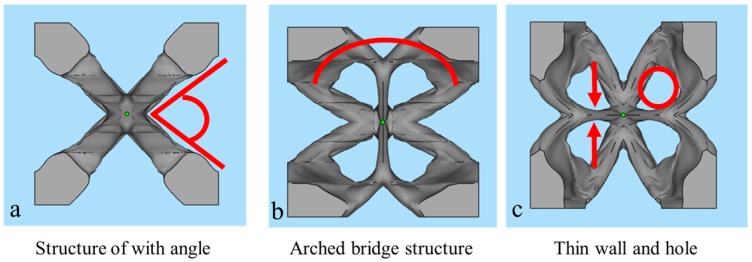
Abstracted typical structures from topology optimized geometries such as structures with angle (**a**); arched bridge structure (**b**); thin wall and small holes (**c**).

Just as mentioned above, the material distribution in the topology optimized geometries mainly depends on stress clouds, which makes the geometries complicated and irregular, and more attention should be paid during fabrication with SLM. Three typical structures are abstracted out of these geometries in Figure 1 such as spiral structure (Figure 4a), arched bridge structure (Figure 4b), structure with thin walls and small holes (Figure 4c). Their manufacturing limits are investigated in the following. 

#### 3.1.1. Manufacturing Limits of Spiral Structure

**Figure 5:**
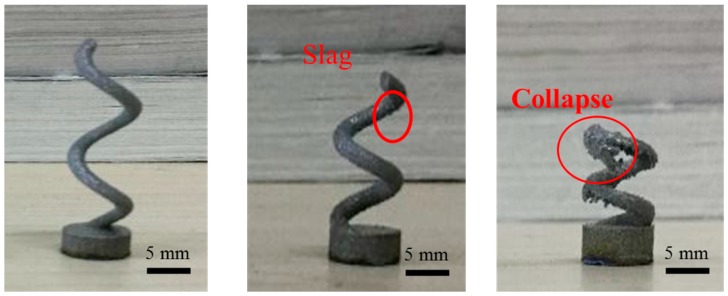
Manufacturing limits and surface finish of spiral structures with rising angle of 60°, 45° and 30°.

Three types of spiral structures with rising angles of 60°, 45° and 30° were investigated, respectively. The outside diameters of the base of spiral structures were 8 mm and that of the spiral line were 2 mm. Three spiral structures were fabricated by SLM without support structures generated using Magics Software. The results are shown in Figure 5.

**Figure 6:**
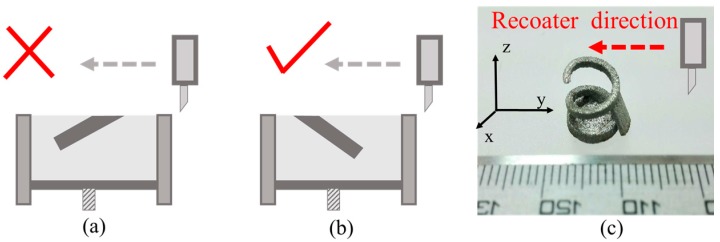
The fiercest (**a**) and weakest (**b**) collision during structure building with a rising angle of 30° and (**c**) manufacturing by SLM after generating support.

By contrast observation, the spiral structures with rising angles of 60° can be fabricated by SLM, and the spiral line has a relatively smooth surface. The structure with a rising angle of 45° also can be fabricated by SLM; however, slags were presented at the lower surface of the spiral line, that worsens the surface finish of the spiral structure. The structure with a rising angle of 30° occurs, exhibiting collapse and cannot be fabricated. Because the structure rises spirally up with the angle of 30°, which always collides with the recoater during the SLM process, the fiercest collision is a head-on one (Figure 6a); when the rising direction of the spiral structure is at a sharp angle of 30° to the direction of movement of recoater, the maximum frictional force between them occurs, and the SLM fabrication process may be stopped. On the contrary, the weakest collision occurs when the rising direction of the spiral structure is at an angle of 150° to the direction of movement of recoater (Figure 6b). It is normally between the maximum and minimum friction values. If there exists unavoidably the fiercest possible collision during the structure building process, the supported structures should be generated for increasing stiffness. Figure 6c shows the manufacturing test specimen of spiral structure with a rising angle of 30° with better surface finish, supporting structure benefits to manufacture.

#### 3.1.2. Manufacturing Limit of the Arched Bridge Structure 

**Figure 7:**
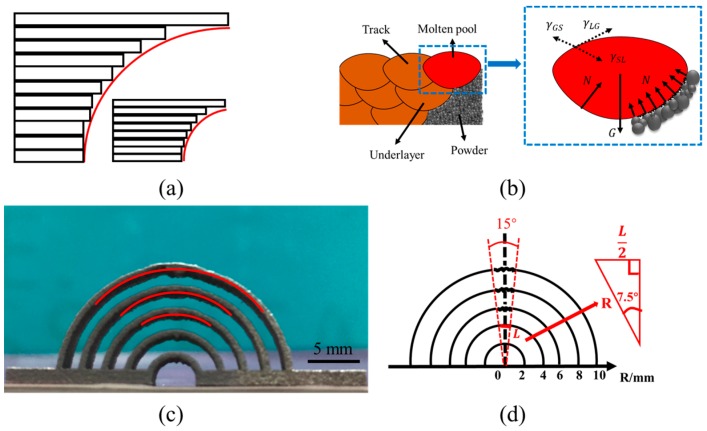
(**a**) Length change of overhangs of arched structures of different radius during SLM process; (**b**) the force acting on the liquid metal in molten pool; (**c**) photos of arched bridge structure manufactured by SLM; (**d**) the collapsed area at the top of the concentric circle varies with different radius of arched bridge structures.

Manufacturability of a series of arched bridge structures with a radius of 2, 4, 6, 8, 10, 12, 14, 16 and 18 mm were investigated. When the radius of the structure is greater than 10 mm, they cannot be fabricated because of collapse. When the structures have a radius of 6, 8, or 10 mm, they can be fabricated, but the surface finish at the top of the arched bridge is worse (Figure 7c). However, the structures with a radius of 2 or 4 mm can be fabricated even with a better surface finish. From the experimental results, the greater the radius of arched bridge structures, the larger the overhang (Figure 7a), and the more it collapses.

As shown in Figure 7b, the force acting on the liquid metal in the molten pool includes surface tension, gravity and powder supporting force from the underlayer during the SLM process. The greater the radius of arched bridge structures, the larger the overhang, then the more liquid metal in the molten pool supported only by powder from underlayer, and not by already solidified tracks. If the gravity of the molten pool is larger than the component of surface tension in the vertical direction, the support by powder from underlayer is not large enough to hold the liquid metal in the molten pool, which penetrates into powder from underlayer and sinter to the lower surface (Figure 7c), seriously worsening the surface quality. Through observation and calculation, the penetration occurs at the top of the concentric circle with radius of 6, 8, and 10 mm, respectively, i.e., it occurs inside the cone with a cone angle of 15°, and the top of the cone is the center of the concentric circle. 

Therefore,

$$L_{max}\approx 2Rsin\theta =2\times 4\times sin7.5{}^{\circ}\approx 1.04 mm$$

here *R* is the largest non-collapse radius, and *θ* is a half of cone angle of collapse area.

If the radius of arched bridge structures is large enough (larger than 10 mm), the gravity of liquid metal in the molten pool is most significant, and the liquid metal of the molten pool sinks directly and the collapse occurs, so the SLM process must be stopped. Therefore, in the phase of structure design for the fabricated geometry, the length of the overhang should be controlled to be less than 1.04 mm for geometries with strict quality requirements, otherwise support structure should be generated by Magics Software.

#### 3.1.3. Manufacturing Limit of Thin Walls and Small Holes

**Figure 8:**
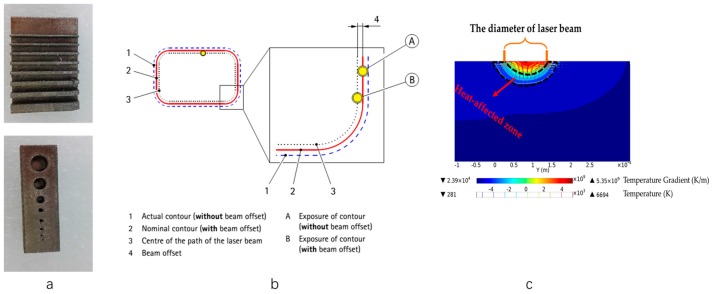
(**a**) Specimens of the thin walls and small holes; (**b**) Beam offset on exposure of the contour; (**c**) Generation of heat affected zone during SLM process.

The manufacturing limit of the structures with thin walls with thicknesses from 0.1 to 1 mm and small holes with diameters from 0.05 to 1 mm was investigated (Figure 8a). The results showed that thin walls with thicknesses less than 0.05 mm and small holes with diameters less than 0.1 mm cannot be manufactured by SLM. Comparison of designed and measured values of thin walls and small holes is listed in Table 1. As can be seen, the measured values of the thickness of thin walls are larger than that of the design values, and the measured values of the diameter of small holes are less than that of design values. The larger the design values, the less the deviation between design value and measured value.

The deviation between design value and measured value depends on the scanning strategies and beam offset value. During the fabrication process of structures with thin walls using SLM (Figure 8b), the laser scans firstly the contour of the thin wall on powder bed and then scans the part of core. The contour line is the centerline of the laser and scanned track. The scanned track is larger than the laser spot size because of the existence of the heat affected zone (Figure 8c), where the powder particles radiated by laser melts as a temperature fields with steep gradient. Thus, the measured values of the thickness of thin walls are always larger than that of the design values, and the measured values of the diameter of small holes are less than that of the design values (Figure 8b). The deviation value between the design and measured value can be adjusted through a change of beam offset value.

### 3.2. Compressive Properties of the Porous Structures

#### 3.2.1. Static Elastic Modulus and Compressive Strength

**Figure 9:**
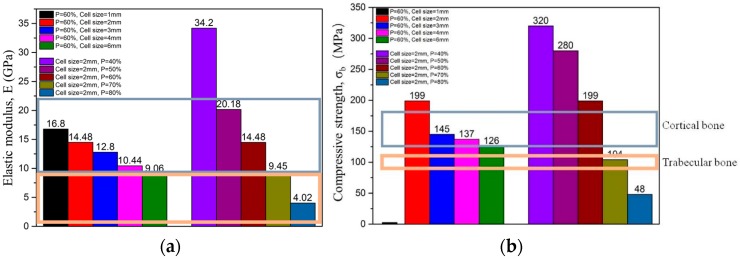
(**a**) Change of static elastic modulus of porous structures with porosity and unit cell size; (**b**) change of compressive strength of porous structures with porosity and unit cell size.

Figure 9 shows the test results for static elastic modulus and compressive strength of two groups of porous structures, one group with a porosity of 60% and unit cell sizes of 1, 2, 3, 4 and 6 mm, and another group with a unit cell size of 2 mm and porosities of 40%, 50%, 60%, 70% and 80%, respectively. It is obvious that both properties tend to decrease with the increase of either the unit cell size (porosity constant) or the porosities (unit cell size constant). The static elastic modulus (*E*) of porous structures measured by compression test are in the range of 4.02 and 34.20 GPa, while the corresponding compressive strengths (*σ_b_*) are in the range of 48 and 320 MPa (the compressive strength of porous structure with *P* = 60% and cell size = 1 mm is comparable to that of solid material). These results are also comparable to trabecular and cortical bones (Ryan and Williams, 1989, Choi et al., 1990, Ashman et al., 1984, Kuhn et al., 1989), which are marked with a grey and yellow color in Figure 9a,b, respectively. In comparison with the Ti-6Al-4V porous structure (*E* in the range of 0.19~6.34 GPa and *σ_b_* in the range of 3.8–112.8 MPa) investigated by Cheng [14], the results suggest that topology optimized porous structure has widened properties shadow than the structure of CAD-based design. 

During the tensile or bend test, specimens can be deformed either by slipping or by local deformation at the loading points, resulting in an underestimated elastic modulus, i.e., static elastic modulus. In general, the dynamic elastic modulus is measured by RFDA, which provides a more credible elastic modulus than the compressive test. Its results will be shown in Table 2 and its relation with stiffness will also be discussed in the following.

#### 3.2.2. Compressive Behavior of Porous Structure

**Figure 10:**
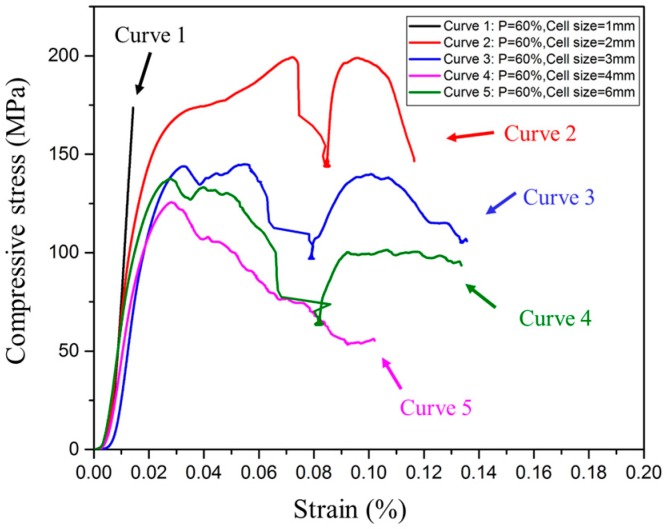
Compressive stress-strain curve of porous structures with a porosity of 60% and different unit cell sizes.

Figure 10 shows the stress-strain curves from the uniaxial compression tests on TOP and SLM fabricated porous structures with a porosity of 60% and different unit cell size. Several investigations showed most of the typical compressive stress-strain curves of porous structures are characterized with three distinct deformation regions, i.e., linear elastic deformation stage, long plateau yielding stage and failure stage; in failure stage it exhibits as sudden and catastrophic collapse [15]. However, the five stress-strain curves obtained from the compression test can be divided into three categories through observation, namely, three failure modes. For the stress-strain curve 1 with black color and unit cell size of 1 mm, it simply exhibits a linear elastic deformation, no stress peak, and no buckling; for the three curves 2–4 with red, blue and purple color, their compressive behaviors comply with the abovementioned failure model, as they are with stress peak and buckling at same time. Whereas for curve 5 with green color, its unit cell size is a little large, its strain increases linearly with stress which it is imposed to, reaches the stress peak, then follows a linear decrease of strain with stress. The curve 5 is only with a stress peak, no buckling. Furthermore, it can be seen that the failure mode of the porous structure is directly related to unit cell size in the case of constant porosity.

**Figure 11:**
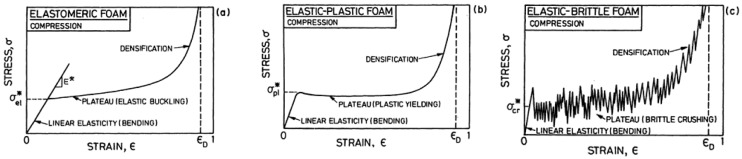
Schematic compressive stress-strain curves for foams showing the three regimes of linear elasticity, collapse and densification: (**a**) for an elastomeric foam; (**b**) for an elastic-plastic foam; (**c**) for an elastic-brittle foam.

Although several investigations were carried out to evaluate the compressive behavior of porous structures, these investigations were scattered, not systematic, and difficultly used as a reference. In order to better explain their compressive behavior, the stress-strain curves for the TOP optimized and SLM fabricated porous structures are discussed based on the deformation mechanism of foam materials. Gibson and Ashby proposed three models of compressive stress-strain curves for elastomeric foam such as rubber, elastic-plastic foam such as metal and elastic-brittle foam such as ceramic, as shown in Figure 11 [7]. Three different models of compressive curves are for different foam materials, but all of them show three similar regimes of linear elasticity at low stresses, long collapse plateau, and a regime of densification, in which the stress rises steeply. Linear elasticity is determined by the bending of the unit cell wall; the static elastic modulus *E* is the initial slope of the compressive stress-strain curve. When the load for compression is continuously imposed, a plateau appears that is associated with the collapse of the unit cell. It exhibits elastic buckling for elastomeric foams, the formation of plastic hinges for elastic-plastic yielding foam and brittle crushing for elastic-brittle foam. When the unit cells almost completely collapse, so that opposing walls of the unit cell touch each other, and further imposed load densifies and strengthens the collapsed unit cells, stress-strain curve increases rapidly at the final region. Although all the compressive stress-strain curves shown in Figure 10 can be divided into three categories, their compressive behavior can be attributed to that of elastic-brittle foam in Figure 11c. In the following, the compressive behavior of porous structures shown in Figure 10 will be discussed based on the elastic-brittle deformation mechanism.

1. Stress-Strain Curve without Stress Peak and Plateau

**Figure 12:**
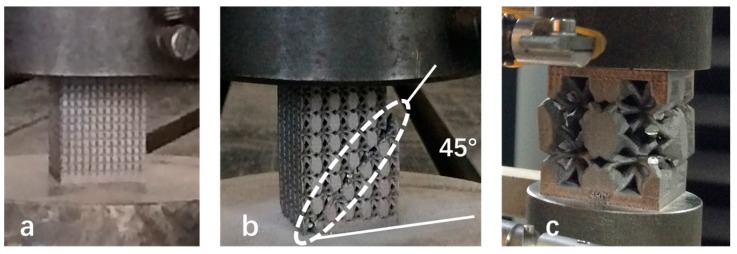
Compressive failure mode corresponding to three categories from compressive stress-strain curves, (**a**) to stress-strain curves in black color without stress peak and plateau; (**b**) to stress-strain curves in blue color with stress peak and plateau; (**c**) to stress-strain curves in green color with stress peak and no plateau.

The stress-strain curve 1 in black color in Figure 10 exhibits that the strain linearly increases with the stress. No stress peak reaches, no plateau appears and no collapse of unit cells (shown in Figure 12a) occurs in the process of stress increase. The loading process is interrupted because of the higher compression resistance of porous structures with small unit cell sizes. The unit cell size of porous structures has a significant effect on compressive strength. With the reduction of unit cell size, the proportion of solid material in porous structure increases and makes the compressive strength of the porous structure more comparable to that of pure solid material. Its compressive stress–strain curve is similar to that of pure titanium reported by Qiang Li [19] and both of them have steeply linearly increasing lines. When the unit cell size of porous structure is less than 1 mm, it can be considered as a pure solid material, and it means that the porous structure with small unit cell size has higher compressive resistance. The stress-strain curve without stress peak and plateau is comparable to the linear elasticity stage shown in Figure 11c. 

2. Stress-Strain Curve with Stress Peak and Plateau

The compressive stress-strain curves 2–4 in red, blue and purple in Figure 10 are characterized with an initial elastic increase, followed by decreasing stress, a plateau with some little oscillations, and finally a short compaction process and complete fracture. All of the three curves show clearly that they have an elastic deformation region with a relatively high degree of linearity up to stress peak. After that, a plateau region appears, in which unit cells collapse due to bucking, yielding and brittle-crushing. Then the failure of porous structures occurs with the increase of strains, the crush bands expanded gradually from the top to bottom, and those bands formed at an angle of 45° with the compressive direction (shown in Figure 12b). Similar crush bands were also observed in typical porous structure [20] and reticulated mesh [14]. The fracture of dense metal normally prefers to occur along the plane which has an angle of 45° with the compressive direction. In addition, a similar tendency of the stress-train curve was also exhibited in porous Ti-6Al-4V produced using a “polymeric sponge replication” method [21]. In short, the porous structures with a porosity of 60% and unit cell size of 2, 3, and 4 mm can be deformed largely experiencing elastic, buckling and yielding deformation before crushing occurs. The stress-strain curve with stress peak and plateau is the same as that in Figure 11c. The difference is that the strain increases with stress after densification but no in Figure 10c.

3. Stress-Strain Curve with Stress Peak and No Plateau

The compressive stress-strain curves 5 in green color as shown in Figure 10 exhibit the compressive behavior of porous structures with unit cell size of 6 mm, that can be distinctly divided into two deformation regions. Firstly, the strain increases with the loading process, a linear elastic deformation occurs in the stage, and then the strain achieves the peak value. After that, the porous structure experiences three continuously developing stages, namely buckling, yielding and crushing. Because the unit cell size of the porous structure is big and the wall of the unit cell is thin, it is not strong enough to resist more deformation. When the strain exceeds the limit, brittle crushing occurs, as shown in Figure 12c. The above failure mode is also reported by Volker, in which the Ti-6Al-4V brittle porous structure is also fabricated by SLM [22]. The stress-strain curve with stress peak and no plateau is comparable to the first wave shown in Figure 11c.

From the above analysis, it can be seen that the compressive strength of the porous structure increases with the decrease of unit cell size. Its deformation behavior and failure mode is directly related to the effect of unit cell size.

### 3.3. Evaluations of the Porous Structures

#### 3.3.1. Measurement of Dynamic Elastic Modulus

The dynamic elastic modulus of porous structures with different unit cell sizes and porosities were measured by RFDA and illustrated in Table 2. The dynamic elastic modulus is inversely proportional to the porosity and the unit cell size, in this view corresponding to the results of the compressive test given in Figure 9. The dynamic elastic modulus is relatively high compared to static results obtained by compression tests, because additional torque is generated in the wall of the unit cell during the compressive test. In addition, the distribution of dynamic elastic modulus marked with red color in Table 2 is also comparable to that of trabecular and cortical bones [23,24,25,26], which ranges wider than that of the porous structures designed by CAD-based, image-based and implicit surface modeling.

#### 3.3.2. Evaluations for Stability of the Porous Structures

Just as shown in Table 2, the dynamic elastic modulus of porous structures can be controlled by the change of their porosity or unit cell size, what provides the possibility to avoid the stress shield phenomenon of presently commonly used surgical implants, and meets well the medical application. However, the stiffness decreases significantly the elastic modulus of porous structures. The stiffness, i.e., deformation resistance, is an important index of the biomechanical properties of materials to evaluate the ability to resist deformation in case of external force. There is no present standard and model for stiffness evaluation for TOP optimized and SLM fabricated metallic porous structures until now. Many models for mechanical properties such as stiffness evaluation for traditional materials such as metal, ceramic or plastic foam have been established based on the Ashby-Gibson model. Qiuyan Li [27] fabricated the random porous structures from PEEK (polyetheretherketone) using die casting, and the relationship between elastic modulus and the relative densities fitted well with the Gibson-Ashby model, with the elastic modulus and mechanical properties matching that of natural human bone through comparison with other porous biomaterials. However, this research was concentrated only on the conformity of their model, and no stability of porous structures with different unit cell sizes was investigated. 

The classical model for open porous structures based on Gibson and Ashby (1997) proposed that the relative modulus (*E/E*_0_) and the relative density (*ρ*/*ρ*_0_) of porous structures satisfy the following relations:

(3) $$\frac{E}{E_{0}}=\lambda {\left(\frac{\rho }{\rho_{0}}\right)}^{n}$$

where *E* is the effective elastic modulus of porous structure, *E*_0_ is the elastic modulus of fully dense material, *ρ* is the density of porous structure, *ρ*_0_ is density the dense material. *λ* is the geometric constant, which can be significantly determined by parameters describing geometrical features such as strut thickness, length and orientation of porous structures, so it is relevant to the biomechanical properties of porous structures. Hagiwara et al. (1987) pointed out that the value of *λ* for open-cell structures from aluminas slightly tends to increase with the increase of unit cell size. More recently, Murr [6] proposed that *λ* can be used to evaluate the functionality of the porous system, for example the stiffness of porous structure influenced by unit-cell dimension.

We try to evaluate the stability of TOP optimized and SLM fabricated porous structures using the value of *λ* in Equation (3). However, the question before us is how to determine the exponent n of power function. In fact, the exponent n of power function in Equation (3), i.e., the classical model for open porous structures based on Gibson and Ashby is generally 2, no matter what material it is. But n will change with the difference in porous structure, and is in the range from 1 to 3. The so-called effective elastic modulus (*E*) for porous structures was calculated using the Equation (3) and let the power exponent *n* to be 2 for biomedical materials such as Ti-6Al-4V. Cheng [14] exhibited that the relative modulus and strength follow a linear relation with the relative density (*ρ*/*ρ*_0_)*^n^* of open cellular structures from Ti-6Al-4V, where *n* is 2.4, which is a little higher than the theoretical value 2 of Gibson-Ashby model. So, to simplify the calculation of geometric constant *λ*, we let n to be 2, combine Equations (1) and (3) and get the Equation (4):

(4) $$\frac{E}{E_{0}}=\lambda {\left(1-P\right)}^{2}$$

It is worth pointing out that Equation (4) suits to materials which porosity *P* > 0.3, which is required by the model. 

**Figure 13:**
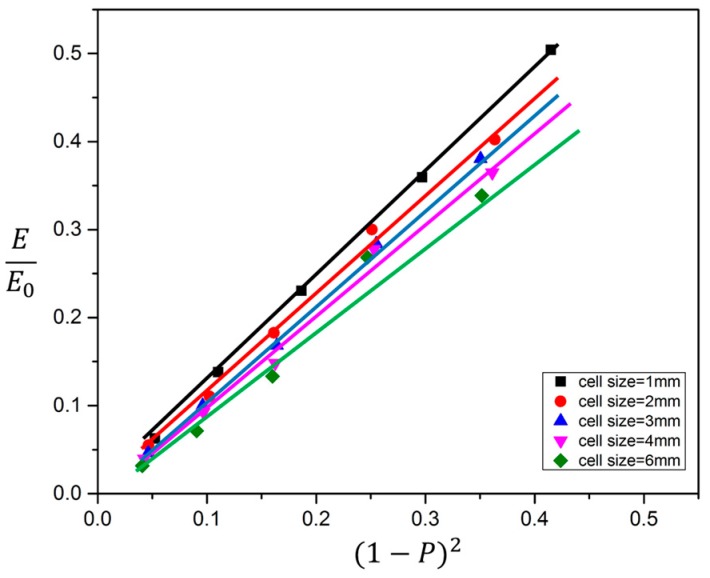
The relative modulus (*E/E*_0_) and the relative density (*ρ/ρ*_0_) of open cellular structures vary with unit cell sizes illustrated in Table 2.

In the following, Equation (4) is considered as the model to evaluate the stability of the porous structure. The relationship of relative modulus (*E/E*_0_) and the square of the relative density (*ρ*/*ρ*_0_)^2^ from data in Table 2 is plotted and shown in Figure 13. The results show that the relative modulus has a linear relation with the square of the relative density, which fits well with the theoretical model of the Gibson–Ashby. The slope of every line is defined as its value *λ*; the greater the value *λ* is, the more stable the structure. It can be seen from Figure 10 that the unit cell size has a significant effect on the stability of porous structures. The less the unit cell size is, the more stable the structure. On the other hand, in combining Figure 10 and Figure 13, it can be seen that the value *λ* of porous structure with unit cell size 4 mm is nearly 1; it is a kind of near elastic material among the elastic-brittle material, its stability balances well with its elastic modulus, and it is also a dividing line for properties of materials. The value *λ* of porous structure is less than 1 when the unit cell size is less than 4 mm. In this case, the stability of the porous structure is worse than its elastic modulus, which is a kind of near elastic material.

### 3.4. Evaluations of the Porous Structures

**Figure 14:**
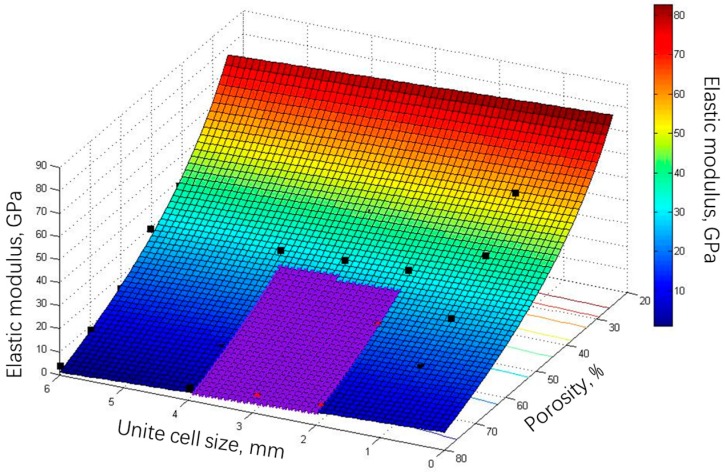
Three-dimensional fitting surface basis on the correlation among unit cell size, porosity and dynamic elastic modulus.

In the above, the biomechanical properties of TOP optimized and SLM manufactured Ti-6Al-4V porous structures are mainly discussed. The elastic modulus and compressive strength (stability) of some porous structures can be controlled by changing either unit cell size or porosity to match that of natural human bone <21.5 GPa, providing the possibility of replacing the missing or defective human bone as well as avoiding the phenomenon of “stress shielding”. According to the design condition and their properties of porous structures, a colorful fitting surface describing the correlation among the elastic modulus, the unit cell size and the porosity of TOP optimized and SLM fabricated porous structure is shown in Figure 14. The elastic modulus in the blue part of the fitting surface in Figure 14 is less than that of natural human bone <21.5 GPa. Referring to the compressive behavior of porous structures with different unit cell sizes, porous structures with the unit cell size 2~4 mm have better compression resistance and stability for implants, and further limit the effective fitting surface for design implants in the purple region shown in Figure 14. The parameters such as unit cell size, porosity and dynamic elastic modulus are calculated by using the data shown in Table 2, and then the Equation (5) about the fitting surface is obtained:

(5) $$E=-87.7892-1.077L+\frac{852.95}{P^{\frac{1}{2}}},$$

where *L* represents unit cell size, *P* represents porosity, *E* represents the dynamic elastic modulus. The equation is obtained by a general-purpose global algorithm with a coefficient *R*^2^ = 0.9944, clearly signifying the correlation among the above three parameters, and is suitable as a mathematical model of the choice of structural parameters for implant design comparable to that of natural human bone. 

It is worth pointing out that elastic modulus *E* in Equation (5) is normally <110 GPa, which is determined by the property of solid Ti-6Al-4V material. Distribution of elastic modulus for natural human bone is different due to age, gender and bone position [15]. For example, for elderly patients with osteoporosis, their mass of bone in the body decreases over time to such an extent that fractures can occur under loads that for young people are considered normal. The natural human bone consists of cortical and trabecular bone, and has a different elastic modulus. Therefore, the above mathematical model is used to choose suitable parameters such as unit cell size and porosity to optimize the elastic modulus and fine control the functions of new implants in the design phase, which is especially for the fitting surface in the purple color region shown in Figure 14. Furthermore, for the fitting surface, namely the mathematical model can be used to design the part with light weight, damping in the industry applications such as aerospace, chemical engineering and sports equipment. At present, the biomechanical properties only for porous structures with a porosity of 60% and different unit cell size were tested, and the difference between the designed and measured elastic modulus and their theoretical law will be investigated based on the current experimental results in the future. Also, the biomechanical properties for porous structures with different porosity and different unit cell size will be simulated and a mathematical model for the full parameters will be developed.

## 4. Conclusions

In order to design and manufacture new medical implants, which matches the elastic modulus of natural human bone, this paper innovatively combines SLM technology with the topology design method. This paper obtains stress clouds under the force condition of the human skeleton using TOP optimization, converts to a special unit cell and constructs a series of porous structures with porosity from 20%, 30%, 40%, 50%, 60% and 80% and unit cell size from 2, 3, 4, 5, 6 and 8 mm, then their manufacturability and structural performance for SLM are investigated. The following conclusions can be drawn.

1. The manufacturing limits of three typical structures abstracted from designed porous structures is given out: the rising angle of spiral structure >30°, the length of overhang from arched structure <1.04 mm, the diameter of hole >0.1 mm, and the thickness of wall >0.1 mm.
2. Properties such as compression strength and dynamic elastic modulus of porous structures fabricated with SLM are measured. The compression test showed the compressive strength and elastic modulus of topology optimized structures can match the requirements of the trabecular and cortical bones of humans. The compressive strength of porous structures decreases with the increase of either unit cell size (porosity constant) or porosity (unit cell size constant). The compressive behavior of porous structures is evaluated and three failure models are proposed based on the Gibson-Ashby model, which can be attributed to that of elastic-brittle foam material.
3. The Ashby-Gibson model was also used to evaluate the stability of porous structures, indicating better stability of the porous structure with smaller unit cell size, which is comparable with the evaluation of compressive strength.
4. The numerical model for correlation among parameters of porous structural and elastic modulus was established, as well as a purple region which was further limited in the effective fitting surface for design implant, indicating the methods for parameter choice during design of porous structure.

At present, the biomechanical properties only for porous structures with a porosity of 60% and different unit cell size were tested; the difference between designed and measured elastic modulus and their theoretical law will be investigated based on the current experimental results in the future. The biomechanical properties for porous structures with different porosity and different unit cell size will be simulated and a mathematical model for full parameters will be developed. In addition, the fatigue properties of materials, as well as biological experiments, including bone ingrowth and osseo-integration, need to be evaluated.

## Figures and Tables

**Table 1:** Measurement parameters of the thin walls and the small holes.

	Thin Wall		Small Hole	
	Designed Value	Measured Value	Designed Value	Measured Value
1	0.05	-	0.05	-
2	0.1	0.16	0.1	0.07
3	0.2	0.24	0.2	0.16
4	0.3	0.33	0.3	0.27
5	0.4	0.42	0.4	0.38
6	0.6	0.61	0.6	0.58
7	0.8	0.81	0.8	0.78
8	1.0	1.06	1.0	0.99

**Table 2:** Dynamic elastic modulus and porosity of porous structures measured by resonant frequency and damping analyzer (RFDA).

Unit Cell Size = 1 mm		2 mm		3 mm		4 mm		6 mm	
*P* ^a^ (%)	*E* ^b^ (GPa)	*P* (%)	*E* (GPa)	*P* (%)	*E* (GPa)	*P* (%)	*E* (GPa)	*P* (%)	*E* (GPa)
35.60	55.47	39.71	44.25	40.8	41.86	39.9	40.13	40.70	37.26
45.50	39.53	49.9	33.01	49.5	31.24	49.7	30.52	50.30	29.52
56.80	25.36	59.83	20.09	59.5	18.53	59.7	16.31	60.00	14.68
66.80	15.17	68.0	12.16	69.0	11.05	68.9	10.19	69.90	7.85
77.10	6.90	78.4	6.07	78.5	5.11	79.4	4.35	79.80	3.50

^a^ Where *P* means the calculated value of porosity based on Equation (1); ^b^ Where *E* is effective elastic modulus of porous structures measured by RFDA.
